# Prevalence of Malaria from Blood Smears Examination: A Seven-Year Retrospective Study from Metema Hospital, Northwest Ethiopia

**DOI:** 10.1155/2013/704730

**Published:** 2013-12-16

**Authors:** Getachew Ferede, Abiyu Worku, Alemtegna Getaneh, Ali Ahmed, Tarekegn Haile, Yenus Abdu, Belay Tessema, Yitayih Wondimeneh, Abebe Alemu

**Affiliations:** School of Biomedical and Laboratory Sciences, College of Medicine and Health Sciences, University of Gondar, P.O. Box 196, Gondar, Ethiopia

## Abstract

*Background*. Malaria is a major public health problem in Ethiopia where an estimated 68% of the population lives in malarious areas. Studying its prevalence is necessary to implement effective control measures. Therefore, the aim of this study was to determine seven-year slide positive rate of malaria. *Methods*. A retrospective study was conducted at Metema Hospital from September 2006 to August 2012. Seven-year malaria cases data had been collected from laboratory registration book. *Results*. A total of 55,833 patients were examined for malaria; of these, 9486 (17%) study subjects were positive for malaria. The predominant *Plasmodium* species detected was *P. falciparum* (8602) (90.7%) followed by *P. vivax* (852) (9%). A slide positive rate of malaria within the last seven years (2006–2012) was almost constant with slight fluctuation. The age groups of 5–14 years old were highly affected by malariainfection (1375) (20.1%), followed by 15–29 years old (3986) (18.5%). High slide positive rate of malaria occurred during spring (September–November), followed by summer (June–August). *Conclusion*. Slide positive rate of malaria was high in study area. Therefore, health planners and administrators should give intensive health education for the community.

## 1. Background 

Malaria is a life-threatening infectious disease caused by the protozoan parasite called *Plasmodium.* It is a leading public health problem in Ethiopia where an estimated 68% of the population lives in malarious areas and three-quarters of the total land mass is regarded as malarious [1] with two-thirds of the country's population at risk [2]. This makes malaria the number one health problem in Ethiopia with an average of 5 million cases per year [3]. The disease causes 70,000 deaths each year and accounts for 17% of outpatient visits to health institutions [4].

Four main species of malaria infect humans: *Plasmodium falciparum *(*P. falciparum*), *P. malariae*, *P. ovale*, and *P. vivax*. *P. falciparum *is the most highly virulent species and is responsible for almost all of the 1.7–2.5 million deaths worldwide caused by malaria [5, 6]. Malaria mostly affects children under the age of 5 years and pregnant women in developing countries [7]. Pregnant women are more vulnerable because they experience depressed immunity during pregnancy, endangering the lives of both mother and the child [8]. A similar problem arises with children below the age of five as their immunity systems are not yet fully developed. It is estimated that every 45 seconds a child dies of malaria worldwide [9].

Malaria is seasonal in most parts of Ethiopia, with variable transmission and prevalence patterns affected by the large diversity in altitude and rainfall with a lag time varying from a few weeks before the beginning of the rainy season to more than a month after the end of the rainy season [10, 11]. Epidemics of malaria are relatively frequent [12] involving highland or highland fringe areas of Ethiopia, mainly areas 1,000–2,000 m above sea level [1]. Malaria transmission peaks biannually from September to December and from April to May, coinciding with the major harvesting seasons.

The main malaria control strategies in Ethiopia include: early diagnosis and prompt treatment, selective vector control, epidemic management, and control, environmental management and personal protection through the use of insecticide-treated bed nets [13]. Despite recent efforts to control the disease, malaria remains the leading cause of mortality and morbidity in the country [1]. A major challenge for malaria epidemiologists is to evaluate the strengths and weaknesses of both methods in estimating malaria incidence and time trends, especially as malaria control programmes are intensified worldwide [14].

Due to the difference in altitude and rainfall, Ethiopia has a varied pattern of malaria transmission, with transmission season ranging from less than three months to more than six months duration [10, 11]. Farming is extensive in study area due to the fact that many daily laborers move from other areas to Metema. Therefore, this study was initiated to analyse seven years, hospital records which are important sources of malaria data, because they are readily available and can provide useful indicators on the situation of malaria at lower cost. Moreover, they are useful to evaluate the impact of the current national malaria control activities on malaria prevalence in the study area. If properly utilized, this information will urge the decision makers to act timely to strengthen malaria control interventions effectively and efficiently.

## 2. Methods

### 2.1. Study Area

The study was conducted at Metema Hospital, which is located in the North Gondar, on the border with Sudan, Amhara region, 897 km North of Addis Ababa and 197 km from the ancient city of Gondar and it has a latitude and longitude of 12°58′N 36°12′E with an elevation of 685 meters above sea level. Metema area is one of the areas where extensive farming is going on in Ethiopia. This area is malarious and it has the only one primary hospital in the community which provides inpatient and outpatient services for more than 5581 populations surrounding it.

### 2.2. Study Design

A retrospective study was conducted to determine the seven years (September 2006 to August 2012), slide positive rate of malaria by reviewing blood film malaria reports at Metema Hospital.

### 2.3. Study Population and Data Collection

The study participants were all malaria suspected individuals who had a complain of febrile illness at Metema Hospital during the study period. Sociodemographic and laboratory data were collected from patients registration book. In this hospital, peripheral smear examination of a well-prepared and well-Giemsa stained blood film is used as the gold standard in confirming the presence of the malaria parasite as WHO protocol. In Ethiopia, the staining techniques and blood film examination for malaria parasite detection were conducted according to a standard operating procedure (SOP) in each hospital and health center throughout the country.

### 2.4. Data Analysis

Data was entered and analyzed using SPSS version 20 software. Chi-square test was employed to compare the proportion and *P* value ≤ 0.05 was considered statistically significant.

### 2.5. Ethical Clearance

The data was collected after ethical clearance was obtained from the School of Biomedical and Laboratory Sciences, College of Medicine and Health Science, University of Gondar. After discussing the objective of the study, written permission was obtained from Metema Hospital before the data collection.

## 3. Results

During the study period, a total of 55,833 malaria suspected patients gave blood films for malaria diagnosis in Metema Hospital. Of these, 9486 (17%) study subjects were positive for malaria. The predominant *Plasmodium* species was *P. falciparum *8602 (90.7%), followed by *P. vivax *852 (9%), and mixed infections of *P. falciparum *and *P. vivax *32 (0.3%). Gender had statistically significant association with malaria infection (Tables 1 and 2).

The age groups of 5–14 years old were highly affected by malaria infection 1375 (20.1%), followed by 15–29 years old with the slide positive rate of 3986 (18.5%). Moreover, malaria infection was statistically associated with ages (Table 3).

High slide positive rate of malaria occurred during spring (September–November) 3118 (20.7%), followed by summer (June–August) 2385 (16.6%), winter (December–February) 2130 (16%), and autumn (March–May) 1853 (14.2%). Season had statistically significant association with malaria infection (Table 4).

Slide positive rate of malaria during the study period (2006–2012) was almost constant with slight fluctuation in the study area. A slightly increased number of microscopically confirmed malaria cases were reported in 2006, followed by 2012, but relatively low number of cases was reported in 2007 (Table 5).

## 4. Discussion

Malaria is a major public health problem in Ethiopia. Over the past years, the disease has been consistently reported as the first leading cause of outpatient visits, hospitalization, and death in health facilities across the country [15]. In this study the overall slide positive rate of malaria was 9486 (17%). This result was lower than similar studies done in Ethiopia [16, 17]. This difference might be due to altitude variation and climatological differences that may contribute to a great role for breeding of *Anopheles* vector. The predominant *Plasmodium* species detected was *P. falciparum*, followed by *P. vivax. *This was in agreement with other previous studies [18–23]. But other a studies reported that the most prevalent species was *P. vivax*, followed by *P. falciparum* [24, 25].

During the last seven years, slide positive rate of malaria was almost constant in a study area with minor difference. A slight increase had started during 2006, but slightly decreased, and continuED almost constantly in 2007–2012. The reduction of malaria cases from 2007 TO 2012 concurS with the increased availability of the new effective drug Coartem for the treatment of *P. falciparum* malaria at national and local levels [26]. Other likely reasons for malaria reduction during this period (2007–2012) might be due to the increased attention to malaria control and preventive activities by different responsible bodies [1].

Males were more infected than females, which was statistically significant (*P* < 0.05). This is in line with the other previous studies [16, 27, 28]. The higher prevalence rate might be due to the fact that males engage in activities which make them more prone to infective mosquito bites as compared to females' counterparts which are mostly at home and protected from such infective bites.

In all age groups, malaria was reported in the study area. However, significantly (*P* < 0.05) affected age groups were 5–14 years old, followed by 15–29 years old. This might be associated with their daily activities. Farming is extensive in Metema due to the fact that young daily laborers move to Metema from different areas for application of herbicide and for gathering of crops. Because of high temperature in this area, daily activities are accomplished especially during night. This may expose them to the bite of mosquitoes.

In the study area, malaria was observed in almost every month of the year, although there was significant (*P* < 0.05) fluctuation in the number of malaria cases (Table 4). The highest prevalence of malaria cases was observed during spring (September–November), followed by summer (June–August) and winter (December–February), while low slide positive rate occurred during autumn (March–May). This is in agreement with other studies [16, 29–31]. The occurrence of malaria depends on adequate rainfall and temperature. In areas with a temperate climate, transmission of malaria is commonly limited to months in which the average temperature is above the minimum required for sporogony [32].

In conclusion, findings of this study showed that slide positive rate of malaria was high and statistically significant with sex, age and seasons. Moreover, its transmission peaks from September to December, coinciding with the major harvesting seasons. This has serious consequences for Ethiopia's subsistence economy and for the nation in general. Therefore, health planners and administrators need to give intensive health education for the community and the daily laborers that mobilized around Metema about the control and prevention of malaria.

This study is limited to the data obtained from the patients' health records, being a secondary data; it is liable to disadvantages associated with any secondary data but we are familiar with the data set and the critical analysis in which the data was subjected into make the conclusion valid.

## Figures and Tables

**Table 1:** Overall slide positive rate of malaria in relation to sex at Metema Hospital, Northwest Ethiopia, 2006–2012.

Sex	No. screened	No. Positive	Percentage (%)	*P* value
Males	30379	5470	18	0.001
Females	25454	4016	15.8	
Total	**55833 **	**9486 **	**17**	

**Table 2:** Prevalence of *Plasmodium *species at Metema Hospital, Northwest Ethiopia, 2006–2012.

*Plasmodium spps *	Frequency	Percentage (%)
*P. falciparum *	8602	90.7
*P. vivax *	852	9
Mixed	32	0.3
Total	**9486**	**100**

**Table 3:** Slide positive rate of malaria by age groups in patients who attended at Metema Hospital, Northwest Ethiopia, 2006–2012.

Age group in year	No. screened	Positive	Percentage (%)	*P* value
<1	4132	473	11.4	0.001
1–4	9909	1630	16.4	
5–14	6847	1375	20.1	
15–29	21593	3986	18.5	
30–44	9645	1511	15.7	
≥45	3707	511	13.8	

**Table 4:** Slide positive rate of malaria at different seasons in patients who attended at Metema Hospital, Northwest Ethiopia, 2006–2012.

Seasons	No. screened	Positive	Percentage (%)	*P* value
Spring (Sep–Nov)	15039	3118	20.7	0.001
Winter (Dec–Feb)	13310	2130	16	
Autumn (Mar–May)	13095	1853	14.2	
Summer (Jun–Aug)	14389	2385	16.6	

**Table 5:** Slide positive rate of malaria from 2006–2012 in Metema Hospital, Northwest of Ethiopia.

Year	Malaria		
	No. screened	Positive	Percentage (%)
2006	8412	1864	22.2
2007	7318	1082	14.8
2008	8600	1389	16.2
2009	7700	1251	16.2
2010	7695	1171	15.2
2011	8248	1289	15.6
2012	7860	1440	18.3
